# Is Cell Death Primary or Secondary in the Pathophysiology of Idiopathic Parkinson’s Disease?

**DOI:** 10.3390/biom5031467

**Published:** 2015-07-16

**Authors:** Walter J. Schulz-Schaeffer

**Affiliations:** Prion and Dementia Research Unit, Department of Neuropathology, University Medical Center Göttingen, Georg-August University Göttingen, Robert-Koch-Str. 40, Göttingen 37075, Germany; E-Mail: wjschulz@med.uni-goetingen.de; Tel.: +49-551-39-220707; Fax: +49-551-39-10800

**Keywords:** neurodegenerative disease, α-synuclein, protein aggregate, synaptic dysfunction, Lewy bodies, cell death

## Abstract

Currently, the pathophysiology of idiopathic Parkinson’s disease is explained by a loss of mainly dopaminergic nerve cells that causes a neurotransmitter deficiency. In the final stage of the disease, there is a marked loss of neurons in the substantia nigra. In addition, Lewy bodies can be found in some of the remaining neurons, which serve as the pathological hallmark of the disease. These Lewy bodies are composed mainly of aggregated α-synuclein, a physiological presynaptic protein. Lewy bodies were thought to be the pathophysiologically relevant form of α-synuclein because their appearance coincided with neuron loss in the substantia nigra. In consequence, neuron loss was thought to be the primary step in the neurodegeneration in Parkinson’s disease. On the other hand, the clinical syndrome suggests a synaptic disorder. If α-synuclein aggregation was causally linked to the pathophysiology of disease, α-synuclein pathology should be found at the synapse. As recently demonstrated, one to two orders of magnitude more α-synuclein aggregates are present in presynaptic terminals than in Lewy bodies or Lewy neurites. Degeneration of dendritic spines associated with synaptic α-synuclein aggregates has been shown to occur in human disease. In experiments, using transgenic mice or cell cultures, mild (two- to three-fold) overexpression of α-synuclein caused an altered vesicle turnover and led to a reduction in neurotransmitter release. Different approaches linked these alterations to presynaptic aggregation of α-synuclein. These findings may fundamentally change the pathophysiological concept of Parkinson’s disease: not nerve cell loss, but the synaptic dysfunction of still existing nerve cells should become the focus of attention. From recent findings, it is quite evident that the death of dopaminergic neurons is a secondary event in the pathophysiology of Parkinson’s disease.

## 1. Do Lewy Bodies Cause Cell Death?

The current pathophysiological hypothesis for the disease mechanism of Parkinson’s disease is that the loss of dopaminergic neurons results in the depletion of the neurotransmitter dopamine in the striatum, which in turn causes the motor symptoms bradykinesia, tremor, rigidity and postural instability[1]. In Parkinson’s disease, Lewy bodies are mainly found at predilection sites of neuronal loss, *i.e.*, the substantia nigra and the locus coeruleus. These findings are the hallmark pathology seen in the final stages of the disease [2]. The co-incidence of both neuron loss and Lewy bodies led to the conclusion that Lewy bodies were the pathophysiologically relevant form of α-synuclein and in combination with cell death were responsible for the disease [3].

The incidence of Lewy bodies in brains of asymptomatic individuals increases with advanced age. This raises the question of whether Lewy bodies reflect presymptomatic Parkinson’s disease, as proposed by Dickson *et al.* [4], or are a feature of normal aging [5]. Gibb reported an age-dependent increase in the prevalence of Lewy bodies from 3.8% to 12.8% between the sixth and ninth decade of age. This is an amount that exceeds the prevalence of Parkinson’s disease by about three- to six-fold [6]. Many other studies show similar findings (for review see [7]).

Because the number of Lewy bodies in patients with mild to moderate loss of neurons in the substantia nigra was reported to be higher than in patients with severe neuronal depletion, Lewy body-containing neurons have been assumed to be the dying neurons [8]. In contrast, it has been shown recently that neuronal dysfunction and loss of nigra-neurons may precede the Lewy pathology [9]. Tompkins and Hill demonstrated that the presence of Lewy bodies does not predispose substantia nigra neurons to undergo apoptotic cell death to a greater degree than the general population of substantia nigra neurons, and that most neurons that undergo cell death do not contain Lewy bodies [10]. Substantia nigra neurons, whether they contain Lewy bodies or not, are similarly affected, for example, by morphological dendritic abnormalities or biochemical changes, indicating that the neurons in general are involved in the disease process [11,12,13,14].

Consequently, attempts to correlate the density of either cortical or brain stem Lewy bodies with clinical symptoms in Parkinson’s disease and DLB were not successful. Most studies failed to correlate Lewy body density with early onset of disease, disease duration, symptoms at onset, visual hallucinations, delusions, recurring falls, severity of parkinsonism, presence or absence of cognitive fluctuations or cognitive decline [15,16,17,18,19]. The presence of symptoms may be related to the involvement of defined regions as measured by the occurrence of Lewy bodies [20,21,22]. However, in a certain percentage of Parkinson’s patients who developed dementia, no Lewy bodies could be detected in cortical areas or in other areas outside the brain stem [23,24]. These findings indicate that the pathophysiology of neurodegeneration as well as cell death can hardly be explained by Lewy bodies or Lewy body-related cell death. It is most likely that Lewy body formation is a process for detoxification of α-synuclein aggregates located at a harmful site in the neuron [25]. Localization, composition and ultrastructure indicate that Lewy bodies are formed in an aggresome-related process and support the notion that Lewy bodies are a compartmentalization of protein aggregates, which attempts to protect the cell [26].

Physiologically, α-synuclein is a protein localized in presynaptic terminals. It promotes the soluble *N*-ethylmaleimide-sensitive factor attachment protein receptor (SNARE)-complex assembly in the form of a chaperone activity [27], and maintains the size of the presynaptic vesicular pool as well as vesicle recycling [28,29,30,31]. Its function is important for neurotransmitter release [32,33], especially for dopamine [34,35,36,37,38].

## 2. Clinical Findings Suggest Synaptic Pathology of Still-Existing Neurons in Parkinson’s Disease

The clinical symptoms of Parkinson’s disease suggest that a failure of synapses is the pathophysiological mechanism of disease. Tremor at rest, rigidity, akinesia/bradykinesia and postural instability are the four cardinal features in Parkinson’s disease [39]. Akinesia/bradykinesia is assumed to be the result of a disruption of motor cortex activity (for review see [39]). Tremor and rigidity were explained by nigrostriatal dopaminergic deficits. Treatment with dopamine substitution or by inhibiting dopamine degradation with monoamine oxidase inhibitors was the major breakthrough for Parkinson’s disease patients in the previous century [40]. Various *in vivo* imaging studies of synaptic functions in the CNS demonstrated presynaptic neurotransmitter deficiencies in Parkinson’s disease (overview in [41]). All of these findings indicate that the degenerative process in Parkinson’s disease is located at the presynapse [42] and results in a neurotransmitter deficiency syndrome. The slow progress of disease and the decreasing effect of dopamine substitution therapy over time argue for a gradually increasing presynaptic failure that precedes nerve cell death.

## 3. An Approach to Explain Neurodegeneration in α-Synuclein Aggregation Diseases other than by Lewy Bodies and Cell Death

When α-synuclein aggregation is causally linked to the pathophysiology of disease, the aggregation should affect the synapse. As the physiological form of α-synuclein is a presynaptically localized protein, α-synuclein aggregation may start at the presynapse. To test this hypothesis we used paraffin-embedded tissue (PET) blotting, the most sensitive known method for the topographical detection of protein aggregates [43]. For technical reasons, we investigated the synaptic α-synuclein pathology first in cortex samples of DLB patients. As predicted by our hypothesis, we were able to detect throughout the cortex a significant amount of α-synuclein aggregates that appear to be much smaller than Lewy bodies. These micro-aggregates were most dense in the cingulate cortex, and their distribution was identical with that of the synaptic protein synaptophysin, suggesting a synaptic localization [44]. The same can be observed at predilection sites in Parkinson’s disease [45].

The problem in the biochemical analysis of α-synuclein aggregates is that these aggregates are highly insoluble [46] so that a reliable quantification of α-synuclein aggregates by Western blot is impossible [47]. We solved this problem by using a protein aggregate filtration (PAF) assay that quantitatively retained the aggregates and separated them from soluble α-synuclein [47]. Unwanted binding of soluble proteins to the membrane was blocked with an amphiphilic polymer [48]. With this method, we were able to show that the amount of synaptic α-synuclein micro-aggregates exceeds the amount of α-synuclein aggregates in Lewy bodies or Lewy neurites by one to two orders of magnitude [44].

An analysis of subcellular fractions of tissues exhibiting the synaptic pathology showed just where at the synapse the α-synuclein micro-aggregates were localized. Fifty to 92% of the α-synuclein aggregates were found in the synaptosome-fraction that contained detached presynaptic terminals (a portion was released from synaptosomes during preparation). To confirm the presynaptic localization, the synaptosomes were disrupted by hypotonic lysis [49], and the α-synuclein aggregates located inside them shifted in the sucrose gradient. The Lewy body fraction contained only 0.02 to 11% of the α-synuclein aggregates [44].

We assumed that the huge amount of presynaptic α-synuclein micro-aggregates have a pathological impact on postsynaptic dendritic spines. By analyzing pre- and postsynaptic markers, we found a 50% reduction of the presynaptic markers synuclein and synaxin compared to controls [44]. This was previously shown by others in DLB and Parkinson’s disease [41,50]. Looking at postsynaptic markers, an almost complete loss of drebrin was observed. Drebrin is an f-actin-binding postsynaptic protein known to be involved in the formation of dendritic spines [51]. By visualizing the dendritic tree of single cells using Golgi-Cox-Davenport silver impregnation, we found a nearly complete loss of dendritic spines in frontal cortical neurons of DLB patients, whereas in age-matched controls the dendrites were densely packed with spines [44]. Reports of a selective reduction of dendritic spines in Parkinson’s disease suggest that the same pathophysiological changes at the synapse underlie Parkinson’s disease that were shown for DLB. Selective loss of dendritic spines were reported for neurons of the prefrontal cortex and basal ganglia using the 6-hydroxy dopamine model of Parkinson’s disease [52,53] or in reserpine-treated mice [54], as well as in the striatal regions and the substantia nigra in human Parkinson’s disease tissues [14,55,56,57,58]. From neurophysiological studies it is known that the formation of postsynaptic dendritic spines is associated with presynaptic activity. Spine shapes are regulated dynamically by synaptic activity, and changes in shape play an important role in synaptic plasticity. Long-term potentiation induces formation of new dendritic spines and deprivation causes a reduction [59] (for review see [60]).

In conclusion, we suggest an alternative explanation for α-synuclein aggregation-associated neurodegeneration other than Lewy body-related cell death. There are one to two orders of magnitude more α-synuclein aggregates than Lewy bodies located in neuron presynaptic terminals. In contrast to oligomers, these micro-aggregates are detergent insoluble, as was shown by the aggregate filtration assay—and also proteinase K-resistant as shown by the PET blot. A postsynaptic loss of dendritic spines is seen in conjunction with the presynaptic micro-aggregates. The loss of synaptic connectivity may result in neurodegeneration markedly earlier than nerve cell loss takes place.

## 4. Link between Synaptic α-Synuclein Aggregation and Synaptic Failure

Modest, two- to threefold, overexpression of α-synuclein leads to familial forms of Parkinson’s disease or DLB [61]. An elevated α-synuclein level accelerates the development of Parkinson’s disease in a dose-dependent manner [62]. Clinically, the decrease of neurotransmitter release is the striking feature in PD. In this context, it is of special interest that Scott *et al.* have shown in cell culture studies that a two- to three-fold overexpression of human wild-type α-synuclein leads to a marked alteration of presynaptic vesicle morphology and a reduction of the presynaptic proteins that are involved in vesicle turnover [63]. Nemani *et al.* have shown that the modest overexpression of α-synuclein at the synapse results in a reduction of the size of the synaptic vesicle recycling pool and a defect in the re-clustering of synaptic vesicles after endocytosis [64]. By using *in vivo* multiphoton imaging techniques, Spinelli *et al.* were able to detect presynaptic α-synuclein micro-aggregates in mice mildly overexpressing α-synuclein-GFP [65]. These micro-aggregates were detectable even in young (one- and three- to six-month old) mice. Hence, it is most likely that the pathological effects on synaptic vesicles seen by the two research groups mentioned above are the consequences of presynaptic α-synuclein micro-aggregates. A link between presynaptic α-synuclein micro-aggregation and deficits in neurotransmitter release has been also demonstrated in transgenic mice expressing a truncated form of human α-synuclein. These mice show a redistribution of the SNARE complex at the presynapse and a reduction in vesicle exocytosis and dopamine release [66,67]. Lim *et al.* demonstrated in human α-synuclein-transgenic mice that the reduction of presynaptic vesicle proteins in hippocampal mossy fibers is an age-dependent phenomenon and thus probably associated with α-synuclein aggregation. In parallel, proteins that are localized at the synaptic plasma membrane, such as syntaxin and SNAP-25, were not diminished [68]. Hence, the synaptic terminals themselves still appear to be present.

In summary, a variety of animal and cell culture experiments confirm the assumption of a link between α-synuclein micro-aggregation at the presynapse and the synaptic deficit that characterizes the disease.

## 5. Neuronal Dysfunction, Lewy Body Formation and Cell Death

It is quite evident that Lewy pathology and death of substantia nigra neurons are, at best, an indirect marker for idiopathic PD. Milber *et al.* have shown that neuronal dysfunction and even neuronal cell loss may precede the Lewy pathology in the substantia nigra [9]. Neuronal dysfunction, nerve cell loss and Lewy pathology in the substantia nigra of 33 incidental Lewy body-(ILB) and 13 PD patients with different Braak stages of Lewy-pathology were compared. All patients were from the longitudinal prospective Honolulu-Asia Aging Study of risk factors for developing PD or dementia. Before Lewy pathology was detectable by conventional immunohistochemical methods, neuronal dysfunction and cell loss could only be observed in the substantia nigra in ILB at levels that on average do not differ from those of higher Braak stages [9]. Consequently, it is under debate whether current pathological criteria are appropriate for diagnosing the whole spectrum of α-synuclein aggregation-associated PD [69]. Lewy bodies and Lewy neurites are most likely a secondary phenomenon of detoxification of the pathophysiologically relevant form of α-synuclein aggregation—the presynaptic α-synuclein micro-aggregates. Lewy bodies and Lewy neurites may be an indirect indicator of the disease process, but do not necessarily reflect the extent of synaptic pathology. It must be taken into account that the ability of various nerve cell populations to form Lewy bodies may differ. Varying competence in the aggresome-formation of nerve cells may be the reason for the lack of correlation between the amount of Lewy bodies and neuronal dysfunction. Nerve cell loss should not be associated with the α-synuclein aggregate detoxification process of Lewy body formation at the cost of ignoring the synaptic pathology and synaptic dysfunction as the relevant event. The underlying synaptic pathology may be the reason why Milber *et al.* found not only dysfunction of neurons preceding Lewy pathology, but also that nerve cell loss preceded Lewy pathology in the substantia nigra [9].

While no correlation has been found between Lewy body content in the substantia nigra and the decrease of dopamine transporter in the putamen or nigral neuronal loss, a decrease in dopamine transporter and neuronal loss seem to correlate with the total α-synuclein aggregate burden [70]. Dijkstra *et al.* found a correlation between nigral neuron decrease and total α-synuclein aggregate burden [71]. Both findings may reflect the synaptic pathology in PD.

## 6. Animal models in Parkinson’s disease

Is it possible to detect the α-synuclein microaggregation-related pathology in animal models of PD as in humans? Unfortunately, there is some difficulty in modeling Parkinson’s disease as this disease does not occur naturally in animals. Moreover, idiopathic PD (iPD) is one of several forms of parkinsonian syndromes that can occur in humans. Idiopathic PD is characterized not only by a dopaminergic deficit, but also by α-synuclein aggregation. This distinguishes iPD from most of the other forms of parkinsonism. Besides in iPD, parkinsonian syndromes occur in association with α-synuclein aggregation also for example in dementia with Lewy bodies (DLB) and multiple system atrophy (MSA). In addition, parkinsonian syndromes can also occur in association with Tau aggregation, resulting for example in cortico-basal degeneration (CBD), progressive supranuclear palsy (PSP) and frontotemporal degeneration (FTD), or they can occur as vascular disease, as a result of repetitive traumata (dementia pugilistica), as post-encephalitic parkinsonian syndrome or as a consequence of intoxication (Parkinson-dementia complex of Guam, intoxication with contaminated synthetic heroin [72], intoxication with herbicides or as a side-effect from drugs) [73]. These parkinsonian syndromes may show a dopaminergic deficit, but most of them not α-synuclein aggregation.

In the 1970s, dopaminergic degeneration was so prominently the focus of Parkinson research that any depletion of the dopaminergic system was considered to be a PD model [74]. Topical unilateral 6-hydroxy dopamine intoxication proved to be the best manageable model for depletion of the dopaminergic system [75]. After some drug addicts presented a clinical picture almost indistinguishable from PD following self-administration of a synthetic heroin analogue contaminated by 1-methyl-4-phenyl-1,2,3,6-tetrahydropyridine (MPTP), a primate model was established that mimics most but not all clinical and pathological features (for instance, not the Lewy pathology) of idiopathic Parkinson’s disease [76]. The idea behind these intoxication models was that PD is caused by toxic molecules and measures for neuroprotection may overcome the disease. Later, after this approach was proven inefficient, the intoxication models were used to investigate pathways that control cell death [74].

As the predictability of toxin-based animal models for clinical studies of PD has been disappointing, genetic mouse models that express wild type, mutated or truncated human α-synuclein under various promotors were developed [77]. As most of these models show neither a relevant loss of dopaminergic neurons in the substantia nigra nor Lewy pathology in substantia nigra neurons, the prevalent belief is that they do not produce the key features of iPD [78]. It must be kept in mind that the read-out of nearly all PD models—irrespective of whether they are *in vivo* or *in vitro* models—is cell death. However, some mutation models present nigrostriatal changes as a result of striatal dopamine loss, suggesting that nigrostriatal neurons are compromised preceding the death of neuronal cell bodies [77]. These findings may parallel the results from Milber *et al.* in ILB and PD patients that neuronal dysfunction is the first neuropathological change and may reflect the synaptic pathology seen in human iPD [9].

Independently, presynaptic α-synuclein aggregation was observed in three α-synuclein-transgenic mouse models: Tanji *et al.* detected presynaptic proteinase-K resistant α-synuclein deposits in a human A53T mutant α-synuclein-transgenic mouse, in which the modified gene was expressed under the prion promoter [79]. Garcia-Reitböck *et al.* observed an α-synuclein accumulation in presynapses of mice expressing human truncated (1–120) α-synuclein under the ratTH-promotor. In consequence, these mice showed a redistribution of synaptic soluble *N*-ethylmaleimide-sensitive fusion attachment protein receptor (SNARE) proteins and a reduction of dopamine-release, whereas a dopaminergic cell death was not observed [67]. Using serial *in vivo* multiphoton imaging, Spinelli *et al.* were able to detect proteinase K-resistant α-synuclein microaggregates selectively in presynapses that were associated with a decrease in the pre-synaptic vesicle protein synapsin. The mice mildly (two- to three-fold) overexpress human wt α-synuclein fused with eGFP under the hPDGFβ-promotor [65].

In summary, different mouse models mimic the presynaptic α-synuclein microaggregation observed in human iPD and DLB, when they mildly overexpress transgenic α-synuclein. In consequence, the vesicle turnover at the presynapse is disturbed. Typically, these mice non-obviously develop dopaminergic nerve cell death.

## 7. Pathophysiological Cascade Leading to Neurodegeneration in PD/DLB

**Figure 1:**
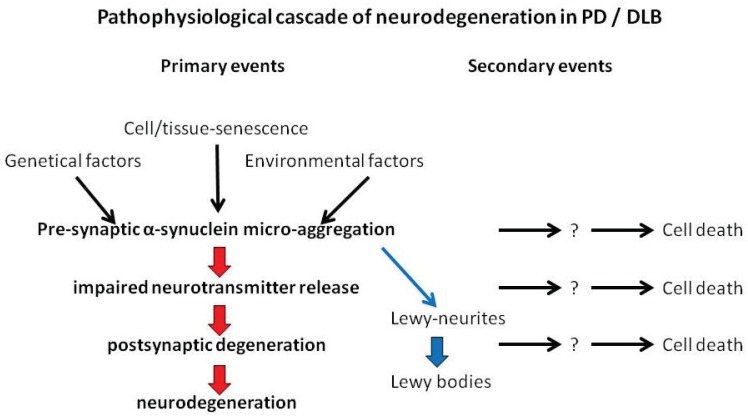
Suggested cascade of events leading to neurodegeneration in PD/DLB.

While the factors responsible for protein aggregation seem to be clear for β-amyloid (β-secretase cleavage of the amyloid precursor protein), Tau (hyperphosphorylation) and prion (conformational change of posttranslationally unaltered protein), the factors leading to aggregation of α-synuclein still remain to be elucidated. Cell and tissue senescence, environmental or genetic factors may contribute to promote the aggregation of physiological α-synuclein (Figure 1). It is most likely that the aggregation process starts at the presynapse. Here, the α-synuclein micro-aggregates influence vesicle trafficking and impair neurotransmitter release. As a consequence, postsynaptic dendritic spines degenerate, and a loss of synaptic connections result in clinical symptoms of neurodegeneration. At the time at which the dendritic spines degenerate, the involved neurons may still be alive (Figure 1).

Several reports suggest that the neurodegenerative process starts at axonal terminals and proceeds in a retrograde direction (review in [80]). At the time of PD diagnosis, the number of neurons with axon degeneration exceeds the nigral neuron loss by 100% [81]. Intra-axonal α-synuclein accumulation occurs as part of Lewy pathology and may impair axonal transport functions.

Only a small percentage of α-synuclein aggregates in Parkinson’s disease are deposited in the form of Lewy bodies [45]. This may reflect the special ability of a subset of neurons to detoxify the noxious α-synuclein micro-aggregates or intra-axonal α-synuclein deposits. There is some evidence that Lewy body formation is an aggresome-related process that segregates excess amounts of unwanted, and possibly cytotoxic, proteins [26].

Neuronal cell death can occur at any time after the neurodegenerative process has been started by presynaptic α-synuclein micro-aggregation (Figure 1). Even the aggregation process itself and not just the deposition of aggregates can be harmful to neurons. This question may be answered in the future by animal experiments. If the impaired nerve cell function can be restored, at least partially, even though synaptic aggregates are present after removal of physiological α-synuclein, then the aggregation process itself might have been the harmful step. When removal of physiological α-synuclein leads to a reduction of presynaptic aggregates, it indicates that neurons may be able to detoxify these aggregates [82]. Micro-aggregates may influence mitochondrial functions or may induce reactive oxidative species (ROS) and the axonal trafficking may be impaired by α-synuclein aggregates, but there is a growing body of evidence that these cell death mechanisms are all secondary events.

## 8. Conclusions

In idiopathic Parkinson’s disease, the aggregation of α-synuclein at the presynapse may be the starting event of the disease process. In consequence, there is a loss of neurotransmitter release and degeneration of postsynaptic dendritic spines. This results over time in the clinical symptoms of neurodegeneration. Some neurons are able to detoxify presynaptic α-synuclein micro-aggregates in an aggresome-related mechanism. In this manner, α-synuclein aggregates are retracted from the synapses through the axons to the cell body forming what we can observe as Lewy neurites and later in the form of Lewy bodies. Cell death may occur at any time during the neurodegenerative process, but is always a secondary phenomenon.
